# Supplementary material for: P-Cadherin Is Expressed by Epithelial Progenitor Cells and Melanocytes in the Human Corneal Limbus
Source: Cells. 2022 Jun 20;11(12):1975. doi: 10.3390/cells11121975 (PMC9221557; doi:10.3390/cells11121975)
Supplement: Supplementary file 1 [file cells-11-01975-s001.zip › cells-1746131-supplementary.pdf]

P-cadherin is expressed by epithelial progenitor cells and melanocytes in the human corneal limbus

Naresh Polisetti[1], Lyne Sharaf[1], Gottfried Martin[1], Günther Schlunck[1], Thomas Reinhard[1]

Supplementary Table S1: Organ cultured corneal scleral tissues used in this study

S.no.	Age (Years)	Post mortem time (h)	Duration of cultivation (days)
1	78	12,08	27,32
2	63	42,17	28,54
3	68	17,15	28,54
4	68	17,15	30,54
5	59	30,25	27,43
6	80	39,93	27,54
7	71	64,52	20,50
8	71	64,52	22,50
9	67	27,67	19,50
10	67	27,67	22,50
11	74	23,87	26,38
12	75	38,50	20,48
13	78	60,63	23,53
14	78	60,63	23,53
15	74	32,17	20,52
16	68	28,92	21,35
17	60	24,17	15,52
18	81	21,92	32,44
19	83	27,48	27,41
20	83	27,48	34,41
21	92	55,33	20,40
22	83	38,17	33,58
23	83	38,17	33,58
24	71	24,73	37,58
25	61	14,13	30,53
26	77	20,00	33,54
27	77	20,00	33,54
28	62	44,50	30,52
29	63	50,65	28,45
30	79	50,72	29,44
31	79	50,72	27,44
32	88	25,50	24,47
33	88	25,50	32,47
34	64	25,07	24,56
35	64	25,07	28,56
36	72	15,48	28,54
37	72	15,48	28,54
38	71	31,88	30,44
39	71	31,88	28,44
40	64	17,33	27,63

41	64	17,33	29,63
42	70	26,72	29,44
43	70	26,72	32,44
44	66	16,43	29,38
45	66	50,42	24,63
46	66	50,42	5,63
47	84	14,33	33,36
48	63	23,37	29,56
49	72	24,83	32,46
50	72	24,83	32,46
51	81	29,42	32,44
52	74	13,45	27,61
53	71	61,18	23,54
54	71	61,18	28,54
55	85	26,33	27,40
56	85	26,33	29,40
57	79	28,33	28,63
58	79	28,33	29,63
59	78	17,33	26,46
60	78	17,33	28,46
61	60	5,58	30,42
62	60	5,58	30,42
63	52	60,68	28,65
64	52	60,68	30,65
65	91	10,25	28,50
66	54	15,82	29,56
67	54	15,82	29,56
68	64	11,75	21,64
69	64	11,75	32,64
70	72	22,98	24,44
71	72	22,98	26,44
72	52	35,08	28,63
73	52	35,08	28,63
74	71	45,43	29,63
75	78	40,75	28,56
76	78	40,75	28,56
77	76	25,92	26,42
78	76	25,92	28,42
79	68	41,67	25,54
80	68	41,67	25,54
81	60	13,67	30,48
82	89	63,58	28,54
83	89	63,58	28,54
84	69	49,93	28,52
85	69	49,93	28,52
86	89	23,10	28,54
87	46	22,00	26,44
88	87	22,12	34,48
89	75	10,87	32,38
90	66	15,83	31,42
91	55	46,85	35,66
92	72	10,95	33,50

93	79	36,80	27,52
94	79	36,80	31,52
95	79	23,75	27,54
96	79	23,75	27,54
97	49	20,90	26,60
98	49	20,90	24,60
99	77	27,77	33,40
100	77	27,77	33,40
101	83	25,60	30,50
102	83	25,60	30,50
103	79	26,03	30,46
104	79	26,03	30,46
105	75	20,67	24,55
106	75	20,67	27,55
107	62	16,33	30,53
108	62	16,33	30,53
109	82	7,33	31,40
110	47	22,50	26,57
111	47	22,50	26,57
112	64	22,42	20,54
113	64	22,42	26,54
114	86	16,50	26,46
115	86	16,50	26,46
116	71	26,17	32,42
117	85	45,38	27,60
118	75	27,67	26,54
119	75	27,67	28,54
120	72	12,68	21,52
121	72	12,68	21,52
122	84	42,80	22,50
123	57	8,25	33,35
124	59	17,12	35,58
125	59	17,12	35,58

Supplementary Table S2. List of antibodies used

**Antibody (clone), Host species**	**Antibody dilution**	**Application**	**Antibody source**
Cadherin-E (24E10), Rabbit	1:200	Immunohistochemistry	Cell signaling
Cadherin-N (D4R1H), Rabbit	1:200	Immunohistochemistry	Cell signaling
Cadherin-P (A-10), Mouse	1:100	Immunohistochemistry	Santa Cruz Biotechnology, INC.
Cadherin-P/Alexa488, Mouse	5 µl/10^6 cells	Flow cytometry	R&D systems
CD90 APC(5E10), Mouse	5 µl/10^6 cells<br>1:400	Flow cytometry<br>Immunohist/cytochemistry	BD Biosciences
CD117 PE (A3C6E2), Mouse	5 µl/10^6 cells	Flow cytometry	Miltenyi Biotec
CD117 PE (YB5.B8), Mouse	5 µl/10^6 cells	Flow cytometry	BD Pharmingen
Cytokeratin pan (PCK-26), Mouse	1:500	Immunohistochemistry	Abcam
Cytokeratin 3/76 (AE5), Mouse	1:100	Immunohisto/cytochemistry	Millipore
Cytokeratin 12 (EPR17882), Rabbit	1:50	Immunohistochemistry	Abcam
Cytokerain 14 (LL002) Mouse	1:500	Immunohisto/cytochemistry	Abcam
Cytokeratin 15 (LHK15), Mouse	1:500	Immunohistochemistry	Abcam
Cytokeratin 15 (EPR1614Y), Rabbit	1:500	Immunohistochemistry	Abcam
Cytokeratin 17/19 (D4G2)	1:50	Immunocytochemistry	Cell Signaling
IgG2a, k, Isotype PE (MOPC-173), mouse	5 µl/10^6 cells	Flow cytometry	Biolegend
IgG3, k Isotype FITC (MG3-35), mouse	5 µl/10^6 cells	Flow cytometry	Biolegend
IgG2a, k Isotype APC (eBM2a), mouse	5 µl/10^6 cells	Flow cytometry	Invitrogen
Melan A, (EPR20380), Rabbit	1:500	Immunohisto/cytochemistry	Abcam
DeltaN p63 (E6Q3O)	1:500	Immunohisto/cytochemistry	Cell Signaling
Vimentin, (D21H3), Rabbit	1:500	Immunohistochemistry	Cell Signaling
Vimentin (280618), Rat	8 µg/mL	Immunohisto/cytochemistry	R&D systems

Supplementary Table S3: Primers used in qRT-PCR primer assays

Gene Symbol	Accession No.	Product Length (nt)	Probe No. /SYBR	Sequence 5 ' – 3'
CDH1	NM_001317186.1	72	P35	CCCGGGACAACGTTTATTAC
				GCTGGCTCAAGTCAAAGTCC
CDH3	NM_001793.5	83	P15	AGGGAGGCTGAAGTGACCTT
				GGGCAGCCCATGAATACTT
GAPDH	NM_002046.5	120	P45	GAGTCCACTGGCGTCTTCAC
				GTTCACACCCATGACGAACA
Ki67	NM_002417.4	78	P39	CCAACCAAAAGAAAGTCTCTGG
				TGATGGTTGAGGCTGTTCCT
ITGA3	NM_002204.3	75	P13	GAGGACATGTGGCTTGGAGT
				GTAGCGGTGGGCACAGAC
KRT3	NM_057088.2	87	P31	TGAGCTGAAGAACATGGAGGA
				TCATTCTCAGCAGCTGTACGTT
KRT15	NM_002275.3	104	P77	TACTTACCGCAGCCTGCTC
				TGATGTGGAAATTGCTGCTG

Primers were used for Probe based (Universal Probe Library) qRT-PCR assays with an annealing temperature of 60°C.
